# The State of the Art in Machining Additively Manufactured Titanium Alloy Ti-6Al-4V

**DOI:** 10.3390/ma16072583

**Published:** 2023-03-24

**Authors:** Chen Zhang, Dongyi Zou, Maciej Mazur, John P. T. Mo, Guangxian Li, Songlin Ding

**Affiliations:** 1 School of Engineering, RMIT University, Melbourne, VIC 3083, Australia; s3458355@student.rmit.edu.au (C.Z.); maciej.mazur@rmit.edu.au (M.M.); john.mo@rmit.edu.au (J.P.T.M.); 2 Faculty of Engineering and Information Technology, The University of Melbourne, Melbourne, VIC 3010, Australia; dzzo@student.unimelb.edu.au; 3 College of Mechanical Engineering, Guangxi University, Nanning 530004, China

**Keywords:** additive manufacturing, machining, cutting force, surface integrity, tool wear, porosity, anisotropy, post-processing processes, hybrid manufacturing

## Abstract

Titanium alloys are extensively used in various industries due to their excellent corrosion resistance and outstanding mechanical properties. However, titanium alloys are difficult to machine due to their low thermal conductivity and high chemical reactivity with tool materials. In recent years, there has been increasing interest in the use of titanium components produced by additive manufacturing (AM) for a range of high-value applications in aerospace, biomedical, and automotive industries. The machining of additively manufactured titanium alloys presents additional machining challenges as the alloys exhibit unique properties compared to their wrought counterparts, including increased anisotropy, strength, and hardness. The associated higher cutting forces, higher temperatures, accelerated tool wear, and decreased machinability lead to an expensive and unsustainable machining process. The challenges in machining additively manufactured titanium alloys are not comprehensively documented in the literature, and this paper aims to address this limitation. A review is presented on the machining characteristics of titanium alloys produced by different AM techniques, focusing on the effects of anisotropy, porosity, and post-processing treatment of additively manufactured Ti-6Al-4V, the most commonly used AM titanium alloy. The mechanisms resulting in different machining performance and quality are analysed, including the influence of a hybrid manufacturing approach combining AM with conventional methods. Based on the review of the latest developments, a future outlook for machining additively manufactured titanium alloys is presented.

## 1. Introduction

Titanium and its alloys are applied in a wide range of industries due to their outstanding mechanical and chemical properties, such as high strength/weight ratio, high elastic modulus, and excellent corrosion resistance. Specifically, the remarkable biocompatibility and good mechanical properties at elevated service temperatures make titanium alloys especially desirable for use in biomedical and aerospace industries, respectively [1,2]. However, the machining of titanium alloys is challenging due to the high strength, low thermal conductivity, and high chemical reactivity of the material [3,4,5]. Due to these properties, it is common to machine titanium alloys with a low cutting speed of less than 90 m/min, which significantly reduces productivity and increases machining costs, when compared to other alloys, such as aluminium, that can be machined with cutting speeds as high as 2500 m/min. The comparatively high feedstock cost of titanium also imposes further economic challenges when machining parts for cost-sensitive applications with high buy-to-fly ratios, which result in large amounts of waste [6,7].

Additive manufacturing (AM) is a new class of manufacturing processes which can produce complex parts directly from a 3D digital model using a layer-by-layer approach with little material waste. Compared to conventional machining processes, such as milling, turning, and grinding in which parts are produced by subtracting redundant materials from feedstock, additively manufactured parts are produced by sequentially consolidating layers of material [8,9,10]. The layered manufacturing approach effectively decomposes a complex and difficult-to-shape 3D part geometry into a series of comparatively simple to manufacture 2D layers. In comparison to conventional machining processes, AM imposes few design constraints on part geometry and requires a low setup effort without the requirement for custom tooling. This allows for the manufacture of complex parts that are highly customisable. AM processes were first commercialised in 1986 through the stereolithography (SLA) process and were only suitable for the manufacture of plastic prototype components [11]. The commercialisation of AM technology for the manufacture of metal components began in the 1990s and has seen extensive development since then [12]. Through continuous development over the past 30 years, it is now possible to build fully dense, functional metallic components with complex geometries for use in demanding industrial applications [13]. For example, as of August 2021, GE Aviation has produced 100,000 additively manufactured fuel nozzle tips for its LEAP jet engines used in Boeing 737 and Airbus 320 [14]. By replacing 855 conventional parts with 12 consolidated components manufactured by AM, GE Aviation has significantly reduced manufacturing process complexity, while also increasing performance, which contributed to a 20% increase in engine fuel efficiency [15].

Despite the advantages of additive manufacturing, metal parts produced by AM processes can exhibit inferior properties compared to wrought machined parts, including higher surface roughness, porosity, dimensional variability, and residual stresses [16,17]. In particular, the high-surface roughness can significantly reduce fatigue life for components subjected to dynamic loading, thereby limiting suitable application areas. As such, additively manufactured metal parts may need to be subjected to various post-processing processes, including machining, in order to obtain acceptable surface quality [18,19], although chemical and electrochemical methods can be applied to improve surface finish of parts with unique geometrical structures, for example, the scaffolds for bone tissue engineering and cellular foams [20,21,22]. A significant number of studies have been carried out on the machining of wrought Ti alloys in the past decades. These studies have focused either on the development of specially designed cutting tools and new tool materials [23,24], optimisation of cutting parameters, application of new cooling media and tribology theories [25], or on the investigation of hybrid machining processes such as thermal-assisted machining (TAM) [26,27,28,29,30]. Since Ti-6Al-4V is the most widely used titanium alloy constituting 50% of total titanium alloy production worldwide, the machining of Ti-6Al-4V has become one of the most widely studied subjects [31,32,33].

The microstructure and mechanical properties of additively manufactured titanium alloys are notably different from their wrought counterparts due to recurring rapid heating and cooling thermal cycles that occur during common metal AM processes (Section 2) [34]. The processing conditions can also vary between specific AM process, resulting in different part mechanical performance, microstructure characteristics, and surface integrity even for the same feedstock material composition [35]. For example, it has been found that Ti-6Al-4V manufactured by Laser Powder Bed Fusion (PBF-LB) (Section 2), the most widely used AM technology, exhibits higher tensile and yield strength than traditionally wrought Ti-6Al-4V due to the presence of higher residual stress and refined α′ martensite resulting from rapid PBF-LB heating and cooling cycles [36]. Similarly, PBF-LB-processed Ti-6Al-4V alloy has been reported to also exhibit higher Young’s modulus [37]. Another factor that drastically affects the mechanical strength of Ti alloys is the oxygen content. Controlling the addition of oxygen, which induces the solution-strengthening phenomenon in the manufacturing process, can improve the mechanical properties of additively manufactured parts [38,39,40]. In additional to differences in machinal properties, the surface roughness, Ra, of PBF-LB manufactured parts (typically 5–40 µm [41]) also differs compared to their machined counterparts (typically 0.4–6.3 µm [42]). The unique properties of PBF-LB parts subsequently affect tool wear, cutting force, cutting temperature, and formation of chips, resulting in significant differences between the machining of additively manufactured and wrought titanium alloys [43,44].

A significant amount of research has been conducted on the microstructure and mechanical properties of additively manufactured Ti-6Al-4V alloys [44,45,46,47,48,49], including studies on their machining characteristics [50,51]. However, there is very limited published literature summarising the influence of mechanical/microstructure properties on the machinability of additively manufactured Ti alloys. Generally, the machinability of materials is influenced by factors including surface integrity, cutting force, cutting temperature, tool wear/life, and chip formation [32,52,53]. Understanding the influence of these factors on the machinability of Ti alloys produced by various AM technologies requires a comprehensive review of the state of the art in several research areas. This paper aims to provide such a review by focusing on the machining of Ti-6Al-4V alloys produced by several Powder Bed Fusion and Direct Energy Deposition additive manufacturing technologies (Section 2). The working principles of these AM processes and how process differences can influence the machinability of additively manufactured Ti-6Al-4V alloys are first discussed. The effects of anisotropy, porosity, and post-processing treatment on additively manufactured Ti-6Al-4V are subsequently reviewed. The mechanisms resulting in different machining performance and quality are analysed. The effects of post-processing and hybrid manufacturing approach on additively manufactured Ti alloys are also reviewed. Finally, based on a summary of the latest achievements, future development trends in machining additively manufactured titanium alloys are presented.

## 2. Additive Manufacturing Processes

The AM process workflow starts with a digital 3D model of the part file, formulated as surface mesh (commonly a stereolithography (STL) file), which is digitally subdivided into individually processable layers for which material deposition or fusion tool paths are generated. During the build process, the tool paths are consolidated in a layer-by-layer approach to produce parts without the need for any custom tooling. The layer-by-layer manufacturing approach effectively deconstructs complex and difficult-to-shape 3D geometry into a series of relatively simpler to shape 2D layers. This key characteristic allows AM to produce parts with unprecedented levels of geometric complexity and design freedom [54,55,56].

A range of AM processes have been developed, which vary in their material feedstock form and material consolidation approach. The most common metal AM approaches are based on Powder Bed Fusion (PBF) and Direct Energy Deposition (DED) techniques [57,58,59]. In a PBF process, a thermal point source scans and selectively melts and fuses the top layer of a bed of atomised metal powder feedstock according to the pre-programmed toolpaths and layers which constitute the desired part. The melt pool rapidly solidifies, the build substrate is lowered by one layer thickness, new powder is deposited, and the next layer is selectively melted. The process repeats until the part build is completed. Following build completion, the unfused powder surrounding the solidified part is removed for reuse in subsequent builds. The ability to rapidly reuse unfused powder in PBF processes provides a significant advantage in material use efficiency compared to conventional subtractive machining processes, which may generate substantial amounts of waste swarf.

**Figure 1:**
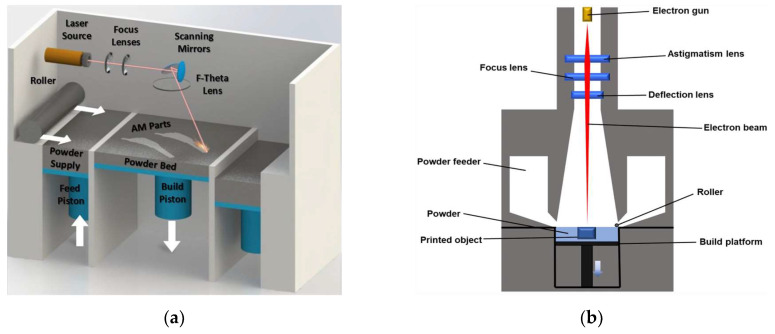

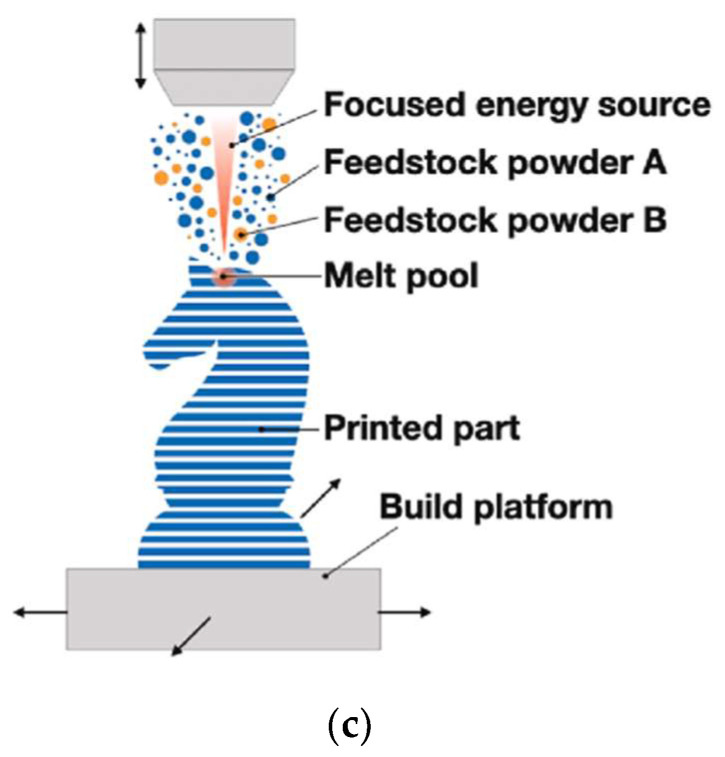
(**a**) Laser Powder Bed Fusion (PBF-LB) process [73] (Reprinted with permission from [73]. Copyright 2018, Elsevier). (**b**) Electron Beam-Powder Bed Fusion (PBF-EB) process [74] (Reprinted with permission from [74]. Copyright 2020, Springer Nature). (**c**) Direct Energy Deposition (DED) process [75] (Reprinted with permission from [75]. Copyright 2022, Elsevier).

In the case of PBF-LB (Figure 1), the irradiating heat source is a high powered, mirror-actuated laser (typically 100–2000 W and ~1 μm wavelength) that is sufficient to melt and fuse the metal powder layers (typically 30–90 μm thick) [60,61]. The representative lasers applied in the PBF-LB process include CO2 laser, Nd: YAG laser (neodymium-doped yttrium aluminum garnet laser), and Yb-fiber laser (ytterbium-doped fiber laser) [60,61,62]. The build chamber is filled with an inert gas (such as argon), and the build substrate onto which the part is built is preheated (preheating temperature can be up to 1200 C [63,64]) to reduce the thermal gradients between the solidifying layers and the substrate. However, the thermal gradients remain high and PBF-LB parts are prone to high levels of residual stress which, in extreme cases, can cause severe part distortion or fracture. Post-build heat treatments are often applied to relieve stresses [65]. The PBF-LB process is also commonly referred to by synonymous trademark terms, such as Selective Laser Melting (SLM) and Direct Metal Laser Sintering (DMLS), that have been popularised by machine manufacturers. However, the terminology standardised by the ASTM International is laser powder bed fusion [66].

The Electron Beam-Powder Bed Fusion (PBF-EB) variant of the PBF process (also referred to as Electron Beam Melting (EBM), Figure 1b) uses an electromagnetically actuated electron beam (typically 3–6 kW) to melt the powder layers (typically 50–150 μm) [67,68]. Compared to PBF-LB, the electron beam allows for higher scanning speeds, powder preheat temperatures, and scan speeds. The higher process temperatures also result in lower thermal gradients and thereby lower residual stress in PBF-EB manufactured parts. However, unlike the PBF-LB, the PBF-EB process needs to take place in a vacuum to avoid E-beam interaction with gas molecules, and this requirement can increase machine cost and setup times [69]. Furthermore, the resolution of manufactured parts is lower than with PBF-LB [70].

Both PBF-LB and PBF-EB technologies allow the creation of parts with small features, high precision, and complex shapes using a variety of alloys. However, these processes involve a range of complex physical phenomena that can impact part manufacturability, quality, and cost, including powder rheology, laser/E-beam absorption in the powder bed, heat transfer, melting and solidification, melt pool dynamics, microstructure development, and thermomechanical stress. The number of influential parameters associated with these phenomena can be large, with estimates suggesting 130 parameters of relevance to PBF-LB [71]. A comprehensive understanding of such influential parameters is the subject of extensive ongoing research [72].

In the DED AM process, feedstock material in the form of powder or wire is fed concurrently with an inert oxidisation-shielding gas (such as argon or helium) through a nozzle, which is coaxial with a thermal source, that melts the feedstock material onto a substrate (Figure 1c). The nozzle is mounted on an articulated robotic arm or gantry system that controls the deposition location of the feedstock material and the layer thickness (typically 200–5000 μm) [76,77,78]. The DED process is generally operated in an inert environment where the oxygen level is controlled to be within less than 5–10 ppm [79,80]. However, some oxidation of the deposited material can occur once the shielding gas is removed from the weld if the part temperature remains high. Unlike PBF processes, the DED process allows for the AM of larger parts or for the repair or refurbishment of existing parts with localised material addition to damaged areas [81,82]. Powder-fed DED processes can concurrently feed different types of powder materials via multiple hoppers, enabling the manufacture of composite materials including functionally graded material (FGM). Variants of DED technology include Laser Metal Deposition (LMD), which uses a laser heat source and can operate with wire and powder feedstock, Electron Beam Additive Manufacturing (EBAM), which uses an E-beam heat source and operates with wire in a vacuum build chamber, and Wire Arc Additive manufacturing (WAAM), which uses an electric arc heat source and operates with wire feedstock. A detailed review of the characteristics of these technologies is available in [83].

## 3. Machinability of Additively Manufactured Ti-6Al-4V

**Figure 2:**
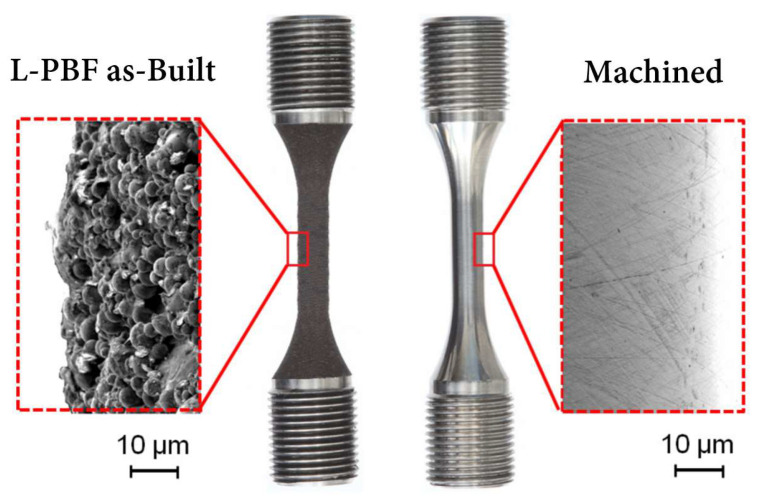
Example PBF-LB surface finish before and after machining process for a Ti-6Al-4V part [90] (Reprinted with permission from [90]. Copyright 2015, Elsevier).

Additively manufactured metal parts typically have a complex, spatially varying thermal history due to the rapid melting and solidification inherent to metal AM processes. This leads to significant differences in residual stresses, surface roughness (Figure 2) and microstructural characteristics (such as grain size and morphology), which results in different mechanical properties compared to conventionally manufactured parts [35,84,85]. Surface roughness is a decisive factor that impacts the mechanical properties of the final products. A high surface roughness can induce stress concentration on the surface and adversely affect the fatigue properties [86]. In general, the surface roughness of additively manufactured Ti alloys is in the range of 10 to 70 μm (Ra), which is significantly larger than that achieved by machining [39,87,88,89]. These differences can also influence the machinability of additively manufactured parts by affecting the cutting force, surface integrity, and tool wear. In order to improve the machinability of Ti-6Al-4V, a comprehensive understating of these influential factors is critically important.

### 3.1. Cutting Forces in Machining Additively Manufactured Ti-6Al-4V

In order to achieve high machining precision, a cutting tool has to follow specially designed tool paths to minimize dynamic changes in cutting load on the tool [91,92]. A sudden change of cutting force can cause poor surface integrity of the workpiece, severe tool wear, and premature tool failure. The cutting force generated by the cutting edge is a significant factor in characterising the machinability of titanium alloys [93]. During the cutting process, the force is exerted on the contact interface between the cutting tool and the chip [94]. Since additively manufactured Ti alloys have a unique acicular microstructure, their strength and hardness can be higher than those of wrought Ti alloys. The higher yield strength can result in a higher cutting force and a high cutting temperature in the cutting zone, which, in turn, will accelerate tool wear rate. The higher hardness can lead to less plastic flow and induce a lower surface roughness [95]. The interaction between the cutting tool and workpiece material, as well as the plastic deformation process during the formation of chips, is affected by the enhanced mechanical properties, the changes of which are finally reflected on the cutting force and surface quality.

Various experimental studies have been conducted to explore cutting force in machining additively manufactured Ti alloys. Polishetty et al. [96] investigated the effect of machining parameters on cutting force and surface roughness of SLM Ti-6Al-4V during the turning process. It was found that higher cutting forces were generated when machining the SLM Ti-6Al-4V alloy due to higher hardness and yield strength. The surface roughness of SLM Ti-6Al-4V was lower because of the high hardness and brittle properties of the material. Shunmugavel et al. [97] found that higher cutting forces when turning SLM Ti-6Al-4V led to higher cutting temperature and tool/chip wear, resulting in significant adhesive/abrasive wear. By comparing the machinability of SLM and wrought Ti-6Al-4V, they found that the cutting force was closely related to the mechanical strength and hardness of the material [18], and the change in these factors was the reason that large cutting forces were recorded when machining SLM Ti [96]. At a cutting speed of 60 m/min, the cutting force was not affected by the tool wear; at 120 m/min, the cutting force increased a little bit when machining both types of parts. However, it rapidly increased when machining SLM Ti alloy at 180 m/min due to the large concentration of wear on the flank face of the insert.

Ming et al. [98] compared the mechanical properties of SLM titanium alloy using different machining processes under dry and minimum quantity lubrication (MQL) conditions. It was found that chip curling was more obvious when milling forged Ti-6Al-4V due to its better plasticity, which indicated that the forged Ti-6Al-4V resulted in a larger main cutting force compared to SLM Ti-6Al-4V. The main cutting force when machining SLM components decreased when the cutting speed was at 80 mm/min due to the thermal-softening effect. However, when machining the forged Ti-6Al-4V, the main cutting force increased slowly. For additively manufactured products, the grain size/morphology is also an indicator of their performance. Coarse columnar grains may be generated in the AM process due to the ultra-high temperature gradient, which results in the anisotropic and poor mechanical properties of additively manufactured products [99,100]. An optimisation of the manufacturing process and alloy composition control has to be conducted to obtain finer equiaxed grains to achieve stronger mechanical properties in the additively manufactured components [101]. Kallel et al. [102] studied the machinability of Ti-6Al-4V produced by Laser Metal Deposition (LMD). Compared to the wrought Ti-6Al-4V parts, the cutting force when machining LMD-manufactured Ti-6Al-4V was 10–40% higher due to their finer grains, which provided higher yield stress and resistance to plastic deformation. The difference in ductility and microstructure (equiaxed grains and lamellar ones) of the wrought parts and the AM parts [35] led to the surface roughness of the LMD parts being 18–65% rougher than that of the conventional samples. The compressive residual stress of the LMD parts was 11–30% higher than that of the wrought samples.

### 3.2. Surface Integrity

**Figure 3:**
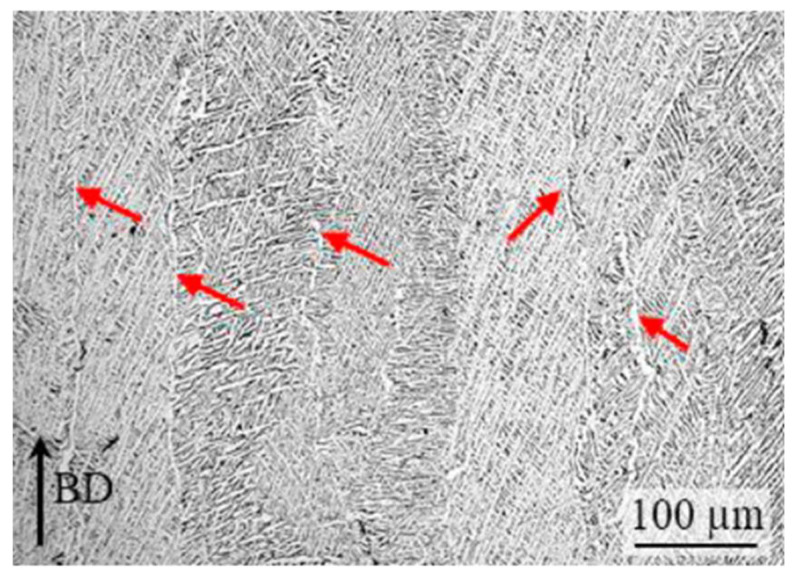
Microstructure along the build-up direction of the PBF-LB Ti-6Al-4V. The red arrows show the α-phase layers along the prior β grains boundaries [103] (Reprinted with permission from [103]. Copyright 2020, Elsevier).

Surface integrity of machined parts is influenced by factors such as machining parameters, cooling conditions, microstructure of the materials, tool wear, and so on. Compared with wrought parts, the surface quality of as-built additively manufactured parts is poor. Lizzul et al. [103] investigated the influence of microstructure on the surface integrity of additively manufactured Ti-6Al-4V fabricated by PBF-LB. According to the analysis of AM-induced microstructure, the material structure, including the α phase layer and the β grains, have a significant effect on the surface roughness of the samples. The AM parameters can impact microstructural anisotropy, which further affects the surface integrity of the as-built parts. The scanning strategy affects the size of prior β grain correlated to the microhardness of the workpiece [104]. Prior β grain boundaries can impact the crack propagation in AM Ti alloy, which has a significant influence on the tensile and fatigue properties of AM Ti parts [105]. The different sizes of β grains of the material are induced by the different thermal histories that have occurred on the fused powder layers. The wider β grains are generated by a lower cooling rate, which leads to thicker α lamellae and lower microhardness. The fracture phenomena occur in correspondence to the α-phase layers that are formed along the β grain boundaries upon heat treatment [38,105]. The α-phase layers formed along the β grain boundaries represent the AM-produced titanium material discontinuity that weakens the material integrity, which benefits material removal during the cutting process (Figure 3). The samples with a minor β grain width exhibit the highest density of discontinuity in the α-phase layers and show a lower cutting force, resulting in a lower surface roughness. The application of the cryogenic cooling strategy can reduce the surface roughness of the final additively manufactured components.

**Figure 4:**
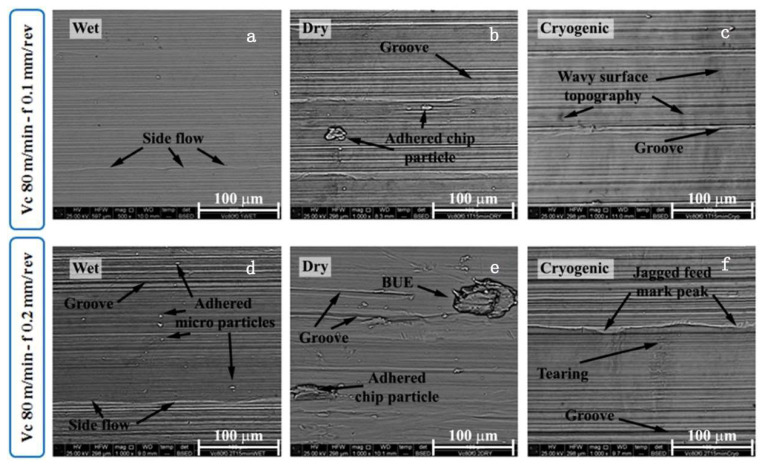
Main surface defects after 8 min of turning when adopting the wet, dry, and cryogenic cooling strategies: (**a**,**d**) material side flow, double feed marks, long grooves, and micro-particles adhered on the machined surface; (**b**,**e**) wider adhered chip fragments and BUE are attached to the machined surfaces; (**c**,**f**) the wavy surface topography along the cutting speed direction and the jagged feed mark peak [107] (Reprinted with permission from [107]. Copyright 2017, Elsevier).

**Figure 5:**
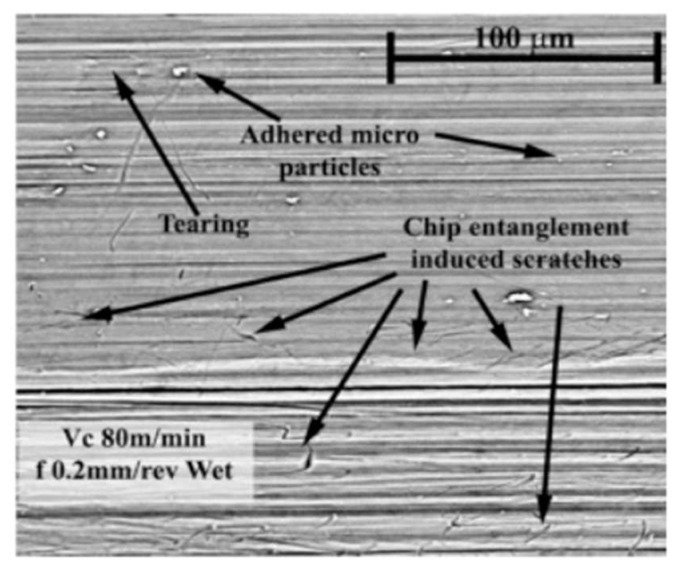
Scratches and adhered particles provoked by chip entanglements after 8 min of wet cutting [107] (Reprinted with permission from [107]. Copyright 2017, Elsevier).

By studying the machinability of wrought, EBM, and DMLS Ti-6Al-4V, Rotella et al. [35] found that the surface roughness of the wrought parts was 10–20% lower than that of the additively manufactured parts. A layer of plastically deformed grains was formed below the machined surface and the thickness of the affected layer, which increased with an increase in cutting speed. The thickness of the affected layer was largest when the cutting speed was 110 m/min. Compared to the wrought workpieces, the EBM parts showed better corrosion resistance and higher surface roughness, and they exhibited higher sensitivity to hardening and compressive residual stress, which was induced by machining, because of their higher hardness. It had been found that cryogenically machined Ti-6Al-4V parts fabricated by EBM had deeper affected layers [106]. The effect of cryogenic cooling was apparent as the thickness of the deformed layer increased on the workpiece surface. However, the plasticity of the material decreased under the cryogenically cooled condition due to the lower cutting temperature, which led to grooved and irregular feed marks on the machined surface. The amount of surface defects increased with an increase in feed rate regardless of the process route. Bordin et al. [107] investigated the surface integrity of Ti-6Al-4V alloy produced by EBM with different cooling strategies, including dry, wet, and cryogenic cooling. It was observed that the cooling condition did not affect the surface quality regardless of the cutting speed when the feed rate was set at the lowest. The use of liquid nitrogen (LN2) reduced the crater and flank wear as well as the surface roughness of the workpiece. Flaws in the machined surfaces, such as side flow, adhesion, tearing, and jagged feed marks, were found; these flaws were affected by the ploughing action between the cutting tool and the workpiece surface. The side flow of the material was caused by the plastic deformation of the surface materials induced by the tool motion. The long straight grooves were caused by the small fragments of the build-up edge (BUE), owing to the rubbing action between the cutting tool and the workpiece. When these fragments were machined through below the flank face of the cutting tool, the grooves were generated on the underlying material; these small fragments left long straight grooves on the material surface when they passed the bottom of the tool flank face. Additionally, adhered materials were left and welded on the surface during the turning process when the adhered particles were formed (Figure 4). Similar surface defects, including side flow and adhered material, can also be found when turning wrought Ti alloy [108]. Cleaner and unbroken surface morphology, as well as randomly oriented micro-scratches induced by chip entanglements, can be observed when the feed rate is 0.1 mm/rev during the wet cutting process (Figure 5).

**Figure 6:**
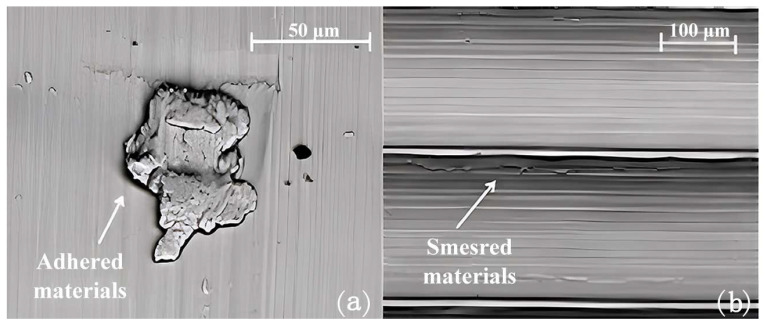
The surface defect generated during dry cutting: (**a**) adhered material and (**b**) smeared material [109] (Reprinted with permission from [109]. Copyright 2016, Elsevier).

**Figure 7:**
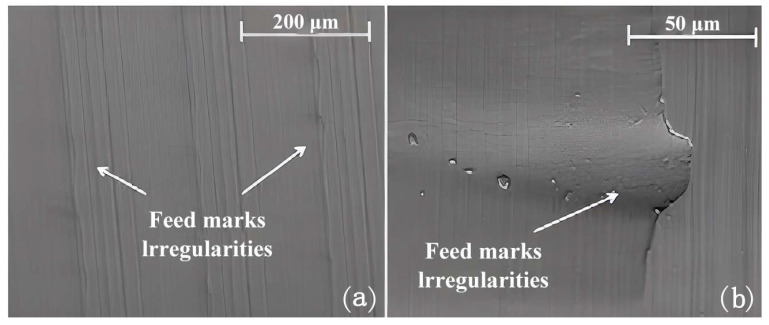
The feed marks and irregularities formed during turning when adopting different cryogenic cooling strategies: (**a**) 500× and (**b**) 2000× [109] (Reprinted with permission from [109]. Copyright 2016, Elsevier).

Sartori et al. [109] studied the machining of DMLS-produced Ti-6Al-4V by using cryogenic cooling during the turning process to improve surface integrity (Figure 6). Compared to dry cutting, a more irregular and jagged surface was produced because of the application of LN2 (Figure 7). The tearing phenomenon was presented on the DMLS part irrespective of the use of cooling strategy. The application of LN2 resulted in the most significant residual stress in the axial direction and induced a thickening of the layer with compressive residual stress.

During the machining process, an ubiquitous compressive stress along the surface of additively manufactured parts could be observed [110]. The cutting region in front of the cutting tool experienced compressive plastic deformation. The compressive stress was generated when the machined surface was squeezed and plastically deformed during the machining process [111]. Oyelola et al. [110] adopted X-ray diffraction to determine the compressive stress in the two measurement directions (axial and circumferential directions) of an additively manufactured Ti workpiece. According to the comparison of the compressive stress in these two directions, it was found that the sample machined with a coated insert incurred higher compressive stresses than the sample machined with an uncoated insert in the parallel direction of the circumference. This difference was induced by the thermal effects associated with chip formation as well as the interactions between the edge of the cutting tool and the machined surface. Furthermore, heat treatment is able to homogenise the microstructure of additively manufactured parts. Coarse microstructures and larger grain size induced by heat treatment lead to a reduction in yield stress, ultimate tensile strength, and hardness of the workpiece, which in turn results in a lower machining force and decreased subsurface deformation, eventually improving the surface roughness [112].

### 3.3. Tool Wear

**Figure 8:**
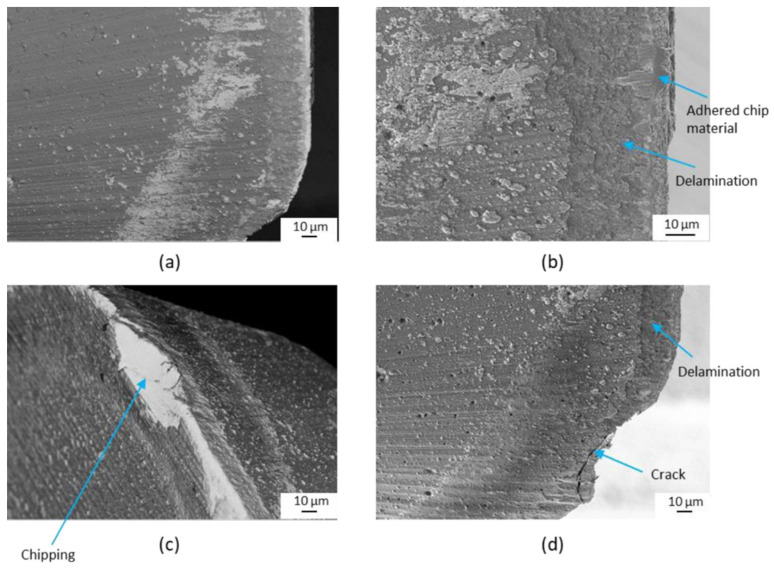
Tool wear mechanisms when machining wrought Ti-6Al-4V alloy: (**a**) SEM micrographs of the cutting tool after a cutting length of 500 m; (**b**) built-up layer of titanium material and coating delamination; (**c**,**d**) fractures and chipping of the cutting edge [121] (Reprinted with permission from [121]. Copyright 2020, Elsevier).

**Figure 9:**
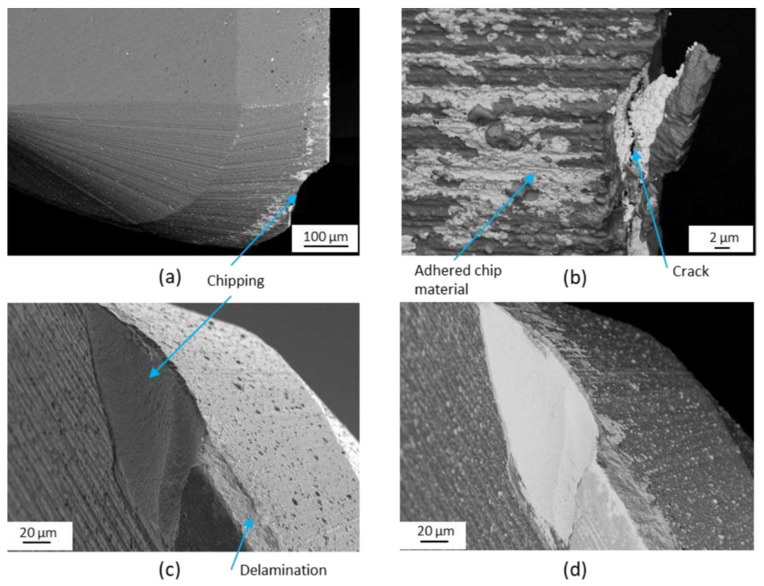
Tool wear mechanisms when machining as-built SLM Ti-6Al-4V alloy: (**a**–**c**) the built-up layer of titanium material, coating delamination, fracture, and chipping of the cutting edge; (**d**) SEM micrograph of the cutting tool after a cutting length of 500 m [121] (Reprinted with permission from [121]. Copyright 2020, Elsevier).

Compared to other metallic materials, titanium alloys are difficult to machine due to their lower thermal conductivity and higher chemical reactivity with the cutting tool. During the machining process, the high cutting temperature and the significant adhesion at the tool–workpiece and tool–chip interfaces can lead to severe tool wear and seriously impact tool life [113,114,115,116,117]. Three dominant types of tool wear mechanisms exist during the machining of titanium alloys: adhesion wear, abrasion wear, and diffusion wear [115,118,119]. Since additively manufactured titanium alloys have different mechanical properties and microstructure in comparison to their wrought counterparts, the effect caused by changes in strength, hardness, and microstructure has to be considered when analysing tool wear when machining additively manufactured Ti alloys. Su et al. [120] studied tool wear when milling SLM Ti-6Al-4V and found that even though the SLM Ti alloy has higher hardness and brittleness as well as lower plasticity than the wrought Ti alloy, severe chip adhesion still can be observed on the rake face and flank face of the cutting tool (Figure 8). Similarly, Al-Rubaie et al. [121] found that regardless of the material features, the major tool wear mechanisms are adhesion, coating delamination, and abrasion. The adhered material, which is caused by the high chemical reactivity of titanium material with the cutting tool, as well as the chips welded on the cutting tool at the interface between the tool and the chips, can be observed on the cutting tool in all machining processes (Figure 8 and Figure 9). The built-up layer of titanium alloy can be seen on all cutting tools. Coating delamination, i.e., the removal of the coating and the exposure of the tool substrate material, can also be seen in the figure.

**Figure 10:**
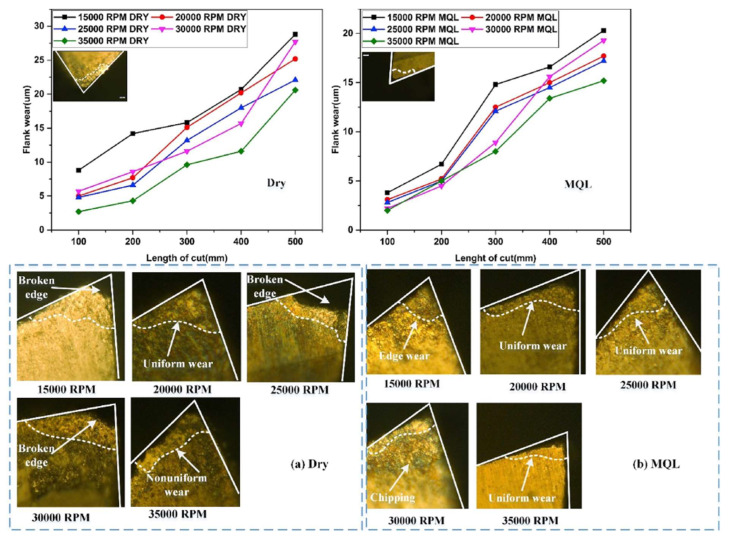
Tool flank wear with increasing feed rate under (**a**) dry conditions and (**b**) MQL coolant conditions [123] (Reprinted with permission from [123]. Copyright 2020, Elsevier).

Minimum quantity lubrication is a new micro-lubrication machining technique that uses an oil mist, rather than a flood coolant, to increase the lubrication capability and reduce the cutting temperature. During the machining process, a mixture flow of cutting fluid and compressed air is atomised and jetted into the cutting area. It can enhance the penetration ability of the cutting fluid and provide significant machining advantages when compared to conventional flood cooling and dry machining in terms of tool life and surface quality [122]. When MQL was used during the machining of SLM Ti-6Al-4V, it was found that abrasive wear was the main reason leading to tool wear due to the adhesion of the cutting tool and the part material [123]. A low cutting speed was unsuitable for machining SLM titanium alloy in the micro-milling process. Specifically, a higher flank tool wear rate was found while using a cutting speed of no more than 55 m/s, and the use of MQL could decrease the flank wear and increase tool life. However, the material adhesion on the tool surface can take off the hard coating of the cutting tool and decrease tool hardness, leading to premature failure of the tool edges, as shown in Figure 10.

**Figure 11:**
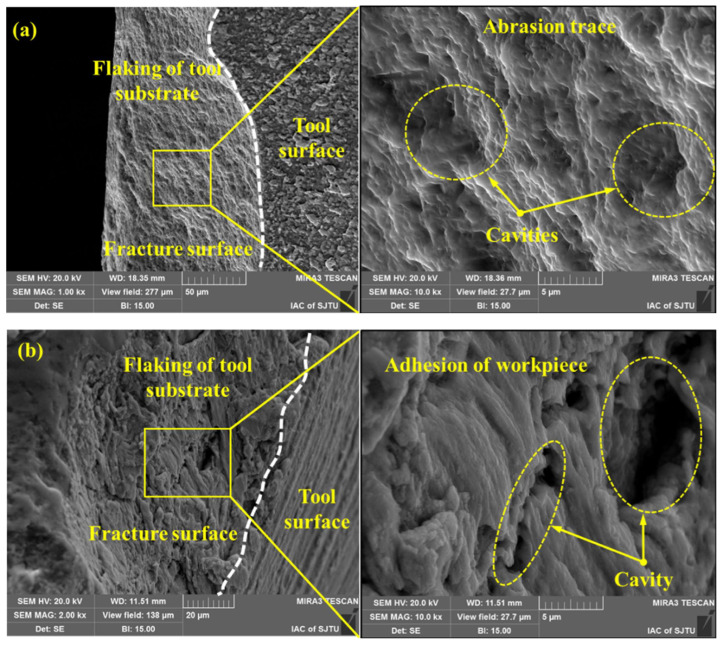
Wear types: (**a**) abrasion marks and (**b**) workpiece adhesion [125] (Reprinted with permission from [125]. Copyright 2020, Elsevier).

During high-speed milling (HSM) of DMLS-built Ti-6Al-4V using ceramic cutting tools, the cutting edge experienced heavy mechanical and thermal loading with the cutting force being up to about 400–500 N and the cutting temperature being approximately 1000 °C [124]; intense friction existed between the chip and the tool, which caused a high temperature around the edge of the cutting tool [125]. Grave wear can be caused on the rake face by a rise in sliding velocity and friction stress [126,127]. The main wear mode of the rake face is adhesion, while both abrasion wear and adhesive wear exist on the flank face (Figure 11). The adhesion of the workpiece material on the rake face and the abrasion on the flank face are adjacent to the tool edge corner, which indicates that diffusion has occurred under the repeated thermal and mechanical loads. The wear is ascribed to the powerful extrusion between the workpiece and the cutting tool, as well as the cyclical robust friction produced by the hard particles of the workpiece. By analysing the degree of tool wear, it has been found that, compared to coated carbide tools, solid ceramic tools can provide better cutting performance during high-speed machining processes [128].

**Figure 12:**
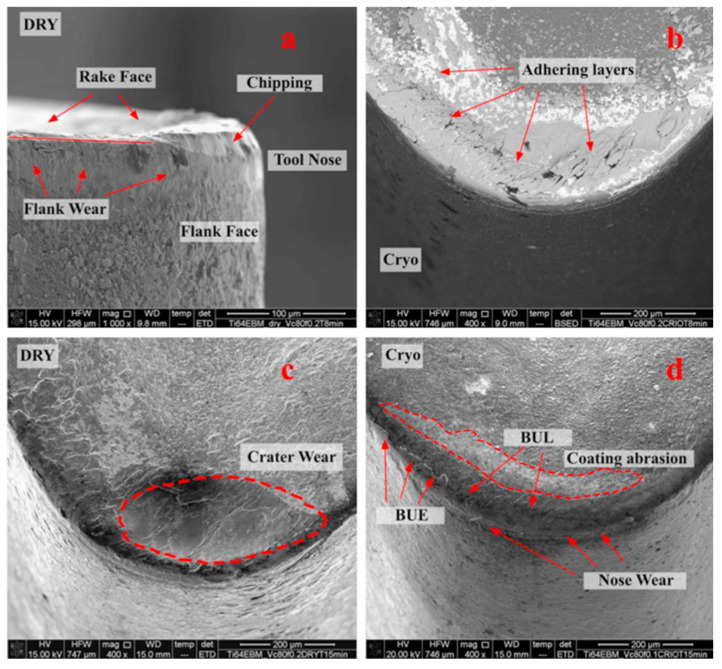
The main tool wear modes observed in dry and cryogenic turning processes: (**a**,**b**) abrasion, chipping, and adhesion on the flank face and (**c**,**d**) crater wear, BUE and BUL on the rake face [129] (Reprinted with permission from [129]. Copyright 2015, Elsevier).

Bordin et al. [129] evaluated tool wear when machining EBM Ti-6Al-4V by adopting coated carbide tools under dry and cryogenic cooling conditions in a semi-finish turning process. It was found that the main wear mechanisms of the flank face are abrasion, chipping, and adhesion of workpiece material (Figure 12a,b). The BUE, built-up layer (BUL), and crater wear can also be seen on the rake face of the cutting tool (Figure 12c,d). Material adhesion is the most crucial wear mechanism during dry and cryogenic machining processes. Chipping is induced by unstable cutting edge fragment under the adhered workpiece material which is welded on the tool surface since the cutting begins. The supply of LN2 in the cutting zone can reduce material adhesion on the cutting edge and prevent the formation of crater wear.

**Figure 13:**
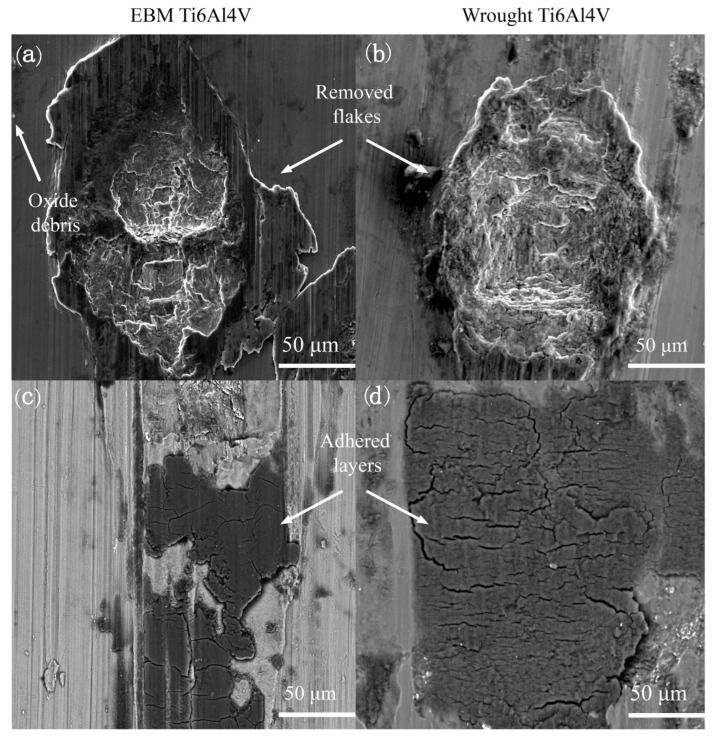
Main wear mechanisms for the EBM and wrought Ti-6Al-4V parts: (**a**,**b**) abrasive wear and (**c**,**d**) adhesive wear [130] (Reprinted with permission from [130]. Copyright 2016, Elsevier).

Abrasive wear and adhesive wear are the primary wear mechanisms when turning EBM Ti-6Al-4V under dry and cryogenic conditions, irrespective of the as-delivered conditions of Ti-6Al-4V, cutting parameters, and cooling strategies [130]. Bruschi et al. [130] found that abrasive wear is presented when wear scars with feed mark peak on the workpiece surface. Due to the high contact pressure, these abrasions lead to the removal of material flakes from the surface together with some titanium oxide debris (Figure 13a,b). These titanium oxide fragments may induce three-body abrasion and increase the wear rate. As shown in Figure 13c,d, adhesive wear appears on the workpiece surface due to the transfer of material, and the appearance of micro-cracks perpendicular to the sliding direction is induced when these layers are subjected to serious deformations. Compared to dry cutting, the cryogenic cooling strategy provides a lower friction coefficient and fewer releases of titanium debris because of abrasive wear.

**Figure 14:**
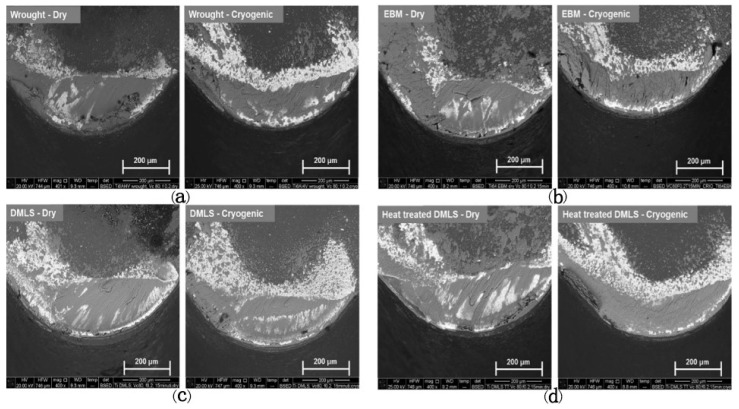
SEM images of worn tool rake faces after 15 min of turning under dry cutting and cryogenic cooling for different Ti-6Al-4V material: (**a**) wrought Ti-6Al-4V; (**b**) EBM Ti-6Al-4V; (**c**) DMLS Ti-6Al-4V; (**d**) Heat treated DMLS Ti-6Al-4V [119] (Reprinted with permission from [119]. Copyright 2017, Elsevier).

**Figure 15:**
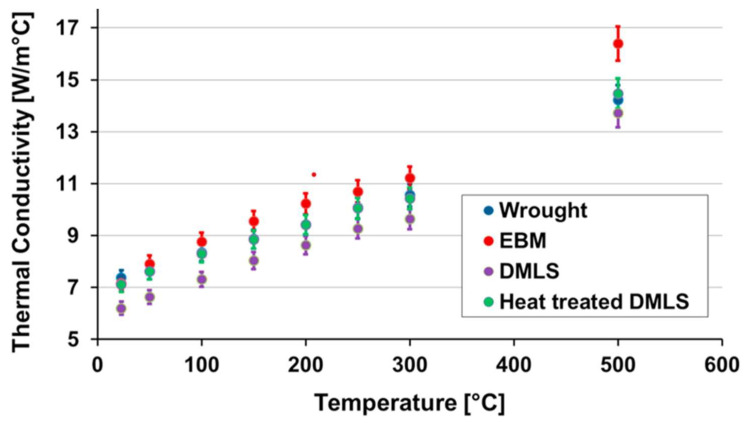
Effect of temperature on the thermal conductivity of Ti-6Al-4V samples under different cutting conditions [119] (Reprinted with permission from [119]. Copyright 2017, Elsevier).

When machining Ti-6Al-4V samples produced by wrought, EBM, DMLS, and heat-treated DMLS, Sartori et al. [119] observed the deepest crater on the tool in the dry-cutting DMLS titanium samples because of their highest hardness, while in cryogenic cooling, the maximum crater depth was reduced to 58% of that in dry cutting due to the reduction in cutting temperature. Figure 14 shows the SEM images of the rake faces of the cutting tool when turning the Ti-6Al-4V under the dry cutting and cryogenic cooling conditions. It can be observed that adhesion and abrasion are the most critical wear mechanisms, irrespective of the as-received conditions and the adopted cutting strategies. In addition, the adoption of LN2 can reduce abrasive wear and flank wear. Based on an analysis of tool wear as well as mechanical and thermal properties, it has been found that EBM parts exhibit the best machinability due to their highest thermal conductivity (Figure 15) and the smallest hardness in comparison to wrought, DMSLed, and heat-treated DMLS parts.

**Figure 16:**
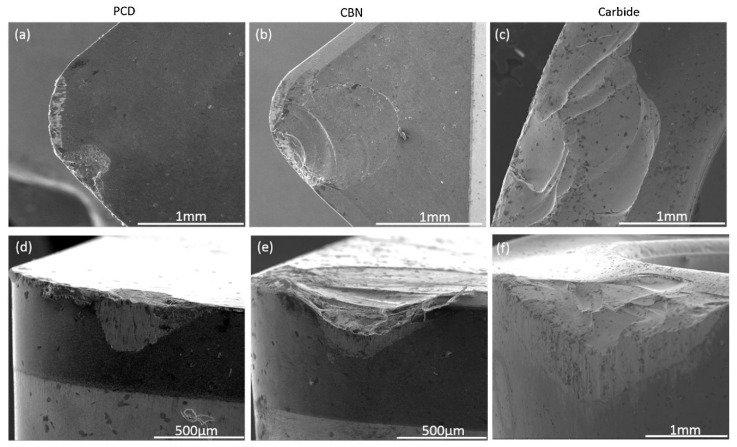
(**a**) Rake face of PCD tool; (**b**) rake face of CBN tool; (**c**) rake face of coated carbide tool; (**d**) flank face of PCD insert; (**e**) flank face of CBN insert; and (**f**) flank face of coated carbide tool [131] (Reprinted with permission from [131]. Copyright 2018, Elsevier).

According to Oyelola et al. [131], the rigid reinforcements and the heterogeneous microstructure in Ti-6Al-4V/WC metal matrix composite (MMC) produced via DED resulted in accelerated tool wear when turning the material with polycrystalline diamond (PCD), cubic boron nitride (CBN) and carbide tools. However, with the outermost layer being removed by subsequent passes, workpiece outrun decreased and tool life was improved. Because less material was pulled out from the MMC surface, the cutting tool experienced less shock impact when the machining process entered the steady state, and abrasion and chipping were found to be the main tool wear modes [132]. The surface integrity was affected by two-body and three-body abrasions induced by the interactions at the cutting region and the pull out of materials. The PCD tool provided the best performance after the outermost layer was removed. When machining with coated carbide tools, WC particles in the MMC acted like tiny cutting edges and grinded the flank face of the tool, resulting in cutting edge abrasion. For both the CBN and PCD cutting tools, the wear around the MMC region resulted in the pull out of material fragments from the cutting tool. These tiny fragments from the cutting tool and those from the workpiece led to accelerated tool wear as the self-abrasive media on the flank face of the cutting edge. On the other hand, the formation of the built-up edge at the cutting edge not only led to adhesion, but also promoted flank wear [133,134]. This phenomenon is most obvious in the CBN tool, as shown in Figure 16.

### 3.4. Chip Morphology

**Figure 17:**
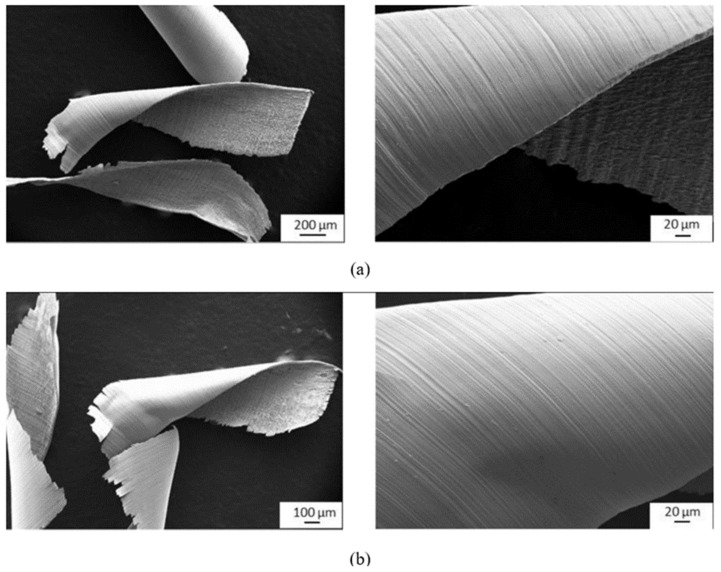
SEM micrographs showing the morphology of chips during the milling process: (**a**) conventional Ti-6Al-4V alloy and (**b**) SLM-formed Ti-6Al-4V alloy [121] (Reprinted with permission from [121]. Copyright 2020, Elsevier).

When machining titanium alloys, the morphology and formation of chips are remarkable indicators of tool wear and level of machinability [28]. The formation of segmented chips is because of the growth of cracks on the chip’s outer surface, or the formation of an adiabatic shear band that is generated by the localised shear deformation as a consequence of the predominance of thermal softening over strain hardening [94,135]. The shear localisation leads to a cyclic change of force (cutting force/thrust) with a sizable magnitude variation. The chatter/vibrations in the machining process can affect the chip and tool wear and restrict the material removal rate (MRR) [94,136]. By examining the chip morphology of wrought and SLM Ti-6Al-4V during the turning process, Coz et al. [137] found that the chip morphologies were similar for the two material states. Helical chips were generated due to the lower uncut chip thickness at a constant feed rate (60 m/min), and a higher cutting speed induced long chips. Additionally, the cutting speed had more significant effect on chip formation during the orthogonal cutting process, which mainly impacted the shear angles, segmentation frequency, and crack length. In micro-scale observations, chips showed irregularly serrated shape in form/frequency, and no particular crack was present. Similarly, Al-Rubaie et al. [121] investigated the generation of chips from conventionally and SLM-produced Ti alloys during the milling processes and found that, despite all the generated chips being fragmented and discontinuous, the chips produced from the SLM workpiece exhibit an increase in the curling degree (Figure 17). This suggests that the chip flow is accelerated in the cutting zone due to minimal frictions. Specifically, the high hardness of the SLM parts tends to reduce the workpiece plasticity and the extent of lateral plastic flow. Therefore, it can reduce the friction at the tool–chip interface and accelerate the chip flow.

**Figure 18:**
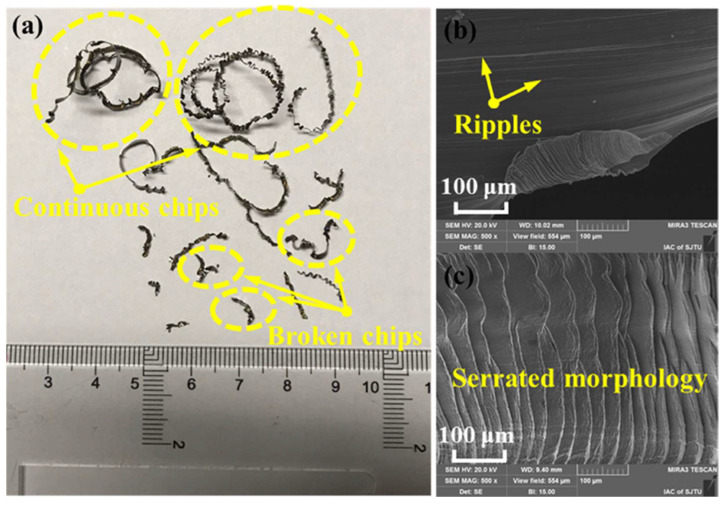
Chip morphologies of DMLS-fabricated Ti-6Al-4V obtained after high-speed milling: (**a**) chip present continuous ribbons and broken pieces, and chip color changes to deep purple in some positions; (**b**,**c**) ripples on the back surface and typically serrated morphology on the free surface of chips [125] (Reprinted with permission from [125]. Copyright 2020, Elsevier).

Zhang et al. [125] observed the chip morphologies of DMLS Ti-6Al-4V in high-speed milling tests. It was found that the chips showed continuous ribbon and broken pieces, and the chips in some positions presented a deep purple colour as a result of the super high cutting temperature causing the burn of the material. According to Figure 18, the typical serrated morphology on the free surface and some ripples that appear on the back surface of the chips can be observed. In the aggressive cutting processes, the high cutting speed generates enough strain to result in the formation of serrated chips.

Furthermore, better control of chips can be conducted when adopting a cryogenic cooling strategy. This is due to the decreased material plasticity because the cutting temperature becomes lower, and the bending capacity and ductility of the material involved in chip formation are reduced [107].

### 3.5. Chatter Vibration

Chatter vibrations are the most frequently encountered problem in the machining of Ti alloys. It is caused by the violent relative motion between the workpiece and the tool because of the instability of the cutting system. Compared to the machining of conventionally shaped Ti workpieces, chatter vibration is more possibly generated during the machining of thin-walled Ti parts due to their low rigidity, time-varying tool–workpiece engagement conditions, and dynamic characteristics [138,139]. Chatter vibration can lead to dynamic interactions, high noise levels, unwanted residual stress, reduced tool life, and poor workpiece surface quality [140]. The mechanism of chatter vibration is still not fully understood because of its complex nature even though it is significantly detrimental to the machining process. The vibrations can affect the fatigue load on the cutting tool and deteriorate tool life. The high vibration magnitude exhibited by additively manufactured Ti parts can be mitigated by post-stress-relief heat treatment, as found by Raval et al. [141]

### 3.6. Mechanical Properties

Although post-processing by using finish machining is required for AM Ti parts to obtain the desired geometrical specifications and surface quality, it is worth noticing that the machining operations can cause machining-induced changes in the mechanical properties of the workpieces. Bertolini et al. [106] found that a highly deformed deformation layer always characterised the machined alloy parts, regardless of the cooling condition and machining route. The cryogenically cooled components showed a deeper affected layer. The EBM-produced samples exhibited a higher sensitivity for machined-induced hardening, and the feed rate significantly affected the compressive residual stresses. In addition, the low cutting temperature reduced material plasticity and induced more grooves on the cryogenically machined surfaces. However, the corrosion resistance grew with the adoption of cryogenic cooling. Ming et al. [98] studied the mechanical properties of SLM Ti-6Al-4V during milling tests and found that high strain rate and temperature could significantly decrease the material flow stress. More energy was consumed during the cutting of SLM parts owing to their low plasticity. The effect of the milling parameter on the residual stress of the SLM parts was not noticeable, but the residual stress of the SLM parts exhibited anisotropy. Furthermore, Oyelola et al. [110] found compressive residual stress on the machined surface of AM Ti parts produced by direct metal deposition (DMD). Umbrello et al. [142] proposed a finite element model to predict the variation in microstructure and nano-hardness of EBM Ti alloy during dry and cryogenic cooling machining processes. Their results showed that a higher cutting speed induced more plastic deformation due to the growth of the shear friction forces at the machined surface. The cryogenic cooling inhibited the strain softening of the surface layers caused by the dynamic recovery, and a higher hardness of the cryogenically machined components could be observed.

### 3.7. Influence of Microstructural Anisotropy

Titanium alloys manufactured by AM technology generally present microstructural anisotropy, which affects their macroscopic mechanical properties [143,144,145,146,147]. The microstructural anisotropy is induced by the unique thermal history at each location during the layer-by-layer building process. Ti-6Al-4V fabricated by the AM process is sensitive to thermal history because the material structure is affected by the temperature and cooling rate [148,149,150]. In general, Ti-6Al-4V manufactured by PBF-LB or PBF-EB exhibits fine acicular, Widmanstätten α–β or martensite alpha’ grains within the prior β phase boundaries [38], which are oriented in the build direction to grow across the building layers during solidification [151,152]. During the AM process, the large columnar prior β grains tend to grow along with the ❬001❭β, which is in parallel with the build direction. The change in the growth direction of β grains leads to the directional solidification texture and anisotropic mechanical properties [79,89,152]. It was reported that horizontally oriented titanium samples provided lower ductility and higher strength than vertically orientated samples [153,154]; however, no apparent difference in tensile strength/yield strength was found [155]. In general, material anisotropy of AM parts is unwanted as it can lead to a fluctuation in cutting force and affect the performance of the final components [156,157].

**Figure 19:**
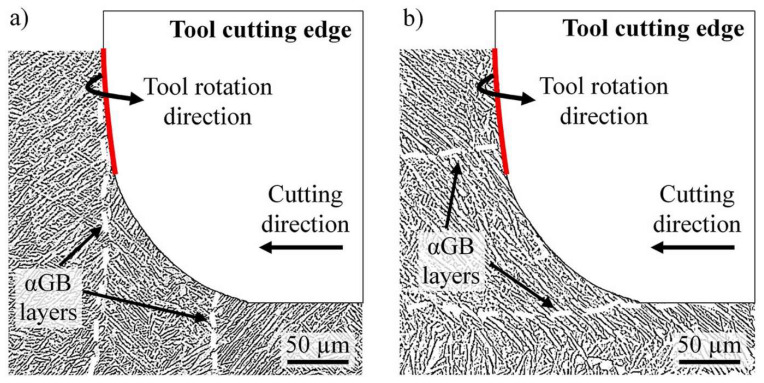
Microstructural interaction when milling 0 deg (**a**) and 90 deg (**b**) oriented PBF-LB Ti-6Al-4V samples [158] (Reprinted with permission from [158]. Copyright 2021, Elsevier).

Lizzul et al. [158] investigated the influence of anisotropy of PBF-LB Ti-6Al-4V parts on the surface quality of the workpiece and chip morphology. The parts were heat treated before the machining was carried out. It was found that α-phase layers (αGB) were present on the prior β grain boundaries. The effect of anisotropy on the machined surface was attributed to the interactions between the α-phase layers and the cutting edge of the tool. The 0 deg and 90 deg samples exhibited similar microhardness (maximum difference was 2%). The αGB layers are positively oriented in relation to the cutting edge when milling the 0 deg Ti samples, as shown in Figure 19. On the contrary, there is no favourable interaction between the αGB layers and the cutting edge in the 90 deg Ti samples. The αGB layers favourably reduced the dislocation movements and the formation of burrs. By analysing chip morphology, it was found a lower cutting force/power was generated when machining the 0 deg Ti samples instead of the 90 deg Ti samples. This phenomenon was more accentuated under the higher cutting speed condition, which prompted the plastic flow of the material and resulted in chips with higher curl radii.

**Figure 20:**
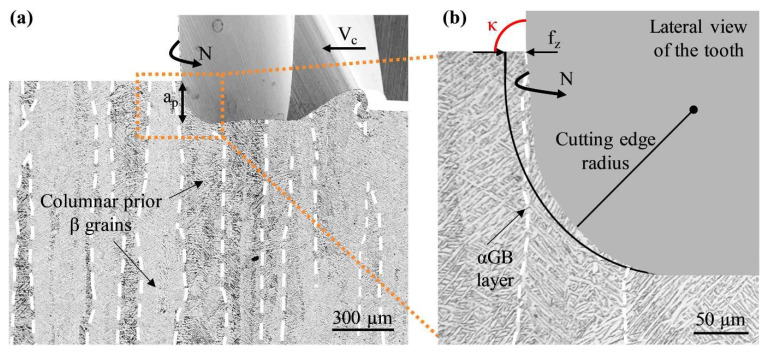
(**a**) Tool engagement with respect to the prior β grain orientations for the 0° sample, and (**b**) zoomed picture showing the orientation angle of the αGB layers relative to the cutting tool registration angle κ [159] (Reprinted with permission from [159]. Copyright 2020, Elsevier).

In another paper, Lizzul et al. [159] evaluated the effect of anisotropy on tool wear when machining Ti-6Al-4V parts produced by PBF-LB with four different build orientations (0°, 36°, 72° and 90°) of αGB layers. Within 1 m of cutting length, the tool diameter was reduced by 3.7% and 7.5%, respectively, when machining the 0 deg Ti sample and 90 deg Ti sample. The tool diameter reduction when machining the 36 deg Ti sample and the 72 deg Ti sample was 5.5% and 6.5%, respectively. During the cutting process, the αGB layer developed along the prior β grains is characteristic of the discontinuity in the microstructure and the weak point along which cracks may develop (Figure 20) [105,150,160]. In addition, when the cutting tool rotates, it gradually engages the workpiece with the registration angle κ (approaches 90°) (Figure 20b). In this case, the orientation angle of the αGB layers corresponds to the registration angle κ for the 0 deg sample, which is favourable to material removal and chip formation, thereby decreasing the cutting force and improving the tool life.

### 3.8. Influence of Porosity

Although additively manufactured Ti parts present excellent surface finish after machining or post-processing, their mechanical characteristics are still affected by the presence of porosity, which results in the deterioration of product quality and a reduction in ductility and strength [45,161,162]. Porosity defects are unavoidable in metal additive manufacturing due to the instability and complexity of the layer-by-layer manufacturing process. The defects can be caused by improper processing parameters, insufficient energy input, and gas entrapped in the powder particles [163], and they can be classified as keyhole pores, lack of fusion pores, and gas pores [164].

During the machining of porous parts, with an increase in machining distance, the spherical pore near the machining path can become tiny and closed, revealing the machining-induced pore closure phenomenon. The presence of pores promotes the dislocation slips and results in the work-hardening effect on the machined surface, in addition to causing changes in cutting force [165]. Ahmad et al. [166] investigated the effect of different porosity on the machineability of additively manufactured Ti-6Al-4V parts through micro-milling experiments. The cutting force was found to have a strong linear correlation with the porosity of additively manufactured parts, and the tool wear increased with an increase in porosity. Micro burrs caused by interrupted chip formation could be observed on the machined surface of the additively manufactured Ti-6Al-4V samples, and the surface roughness increased with an increase in porosity. A similar experiment was conducted by Varghese et al. [167], who investigated the influence of porosity on the cutting force and surface integrity of Ti-6Al-4V alloy produced by DMLS using a micro-milling process. The results demonstrated that the cutting force when machining additively manufactured Ti-6Al-4V with 0% porosity was maximum. It decreased with an increase in porosity and reached the minimal level at 46% porosity. On the other hand, with an increase in cutting depth, the cutting force when machining porous additively manufactured Ti parts increased due to the heterogeneous nature of the porous workpiece material and the non-uniformity in pore size. The surface roughness of the additively manufactured Ti-6Al-4V alloy decreased with the increase in cutting depth. With an increase in porosity, the surface roughness of the additively manufactured part began to increase first and then decreased with the continuous increase in porosity because of the smearing/porosity closure phenomena. Shunmugavel et al. [95] found that there was a significant difference in cutting force when machining conventional Ti parts and additively manufactured Ti parts at a high cutting speed of 180 m/min because of the thermal softening effects and the existence of pores. When machining the SLM Ti component, the pores resulted in micro impacts on the cutting tool, and the frequency of these micro impacts increased with an increase in the cutting speed. Hence, a higher cutting force could be induced during the high-speed machining of the SLM Ti-6Al-4V sample.

### 3.9. Influence of Post-Processing Processes

Ti-6Al-4V parts produced by AM show comparatively high yield stress (about 1000 Mpa) and high tensile strength (around 1150 MPa), but relatively low ductility (smaller than 10%) [168,169]. The high cooling rate during the AM process results in significant internal thermal stresses in the material structure. During the building process, the scanning by the heat resource (laser or electron beam) may lead to instabilities in the melt pool, which, consequently, causes a rise in porosity and surface roughness [55,168]. These features will deteriorate the fatigue performance of the additively manufactured Ti components and impact their reliability in engineering applications. Hence, to improve mechanical properties, reduce porosity, and alter the microstructure of additively manufactured Ti components so as to meet application requirements, besides machining, post-processing methods such as heat treatment and hot isostatic pressing (HIP) are usually applied after the parts are built [79,168,170,171,172,173]. These processes can relieve stresses, minimise porosity [171,174,175,176], and affect the response of subsequent machining processes. Oyelola et al. [112] studied the influence of heat treatment on the machining of DED-fabricated Ti parts. They found that heat treatment can improve the machinability of DED parts due to the homogenising of microstructure. The microstructural homogenisation results in a reduction in the average machining force. The larger grain size generated by the post-heat treatment leads to a reduction in hardness, which improves surface roughness and substantially decreases subsurface deformation. Similarly, Al-Rubaie et al. [121] found that stress-relieved additively manufactured Ti parts exhibited a larger cutting force compared to wrought and as-built parts because the stress-relief heat treatment increased the compressive stress. In another study, Bruschi et al. [177] investigated the influence of heat treatment on tool wear behaviours when machining EBM-built Ti components. They found that the heat treatment had positive influences on tool wear behaviours, and all the heat-treated samples exhibited a lower friction coefficient/tool wear rate because the heat treatment increased the sub-surface hardness of the heat-treated parts through changing the microstructure.

## 4. Hybrid Manufacturing of Additively Manufactured Ti-6Al-4V

Additively manufactured metal parts have poor dimensional/geometric accuracies and poor surface quality because of the “staircase-effect” and non-uniform powder deposition [178,179]. In order to solve these issues, subtractive machining including milling and turning has been extensively applied when post-processing quality-critical components, such as low-pressure turbine blades and blade disks. However, the machining of internal surfaces, which are unreachable by cutting tools, is not possible. This issue has significantly limited the high-level design flexibility of AM and restricted its application in manufacturing functional components with complex shapes.

**Figure 21:**
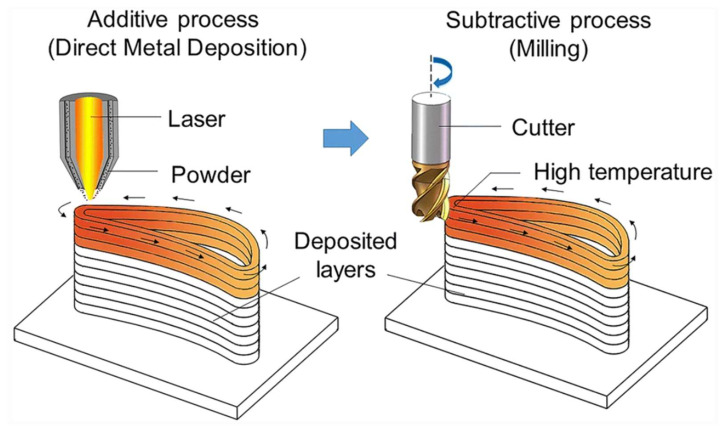
Additive and subtractive hybrid manufacturing processes [180] (Reprinted with permission from [180]. Copyright 2020, Springer Nature).

To solve this bottleneck problem, a new type of “hybrid manufacturing” machine has been developed recently by integrating the machining and AM process together [29,30]. The machining of internal surfaces is conducted during the AM process before the surfaces become inaccessible after the AM process is finished (Figure 21) [180]. Nevertheless, due to the lack of in-depth knowledge of the hybrid process, the strategies used in in situ machining are still those developed for conventional machining, and as a result, serious quality problems, such as defective surfaces and excessive tool wear, frequently occur [181,182,183].

Ye et al. [184] developed a hybrid manufacturing machine tool consisting of high-speed milling and direct metal deposition to acquire additively manufactured products with good surface quality and high precision. The surface quality can be significantly improved due to the removal of the rough layers by the milling process. Jeng et al. [185] presented a hybrid process that combines selective laser cladding (SLC) and the milling process to prove the feasibility of modifying/repairing the parts by applying the hybrid process.

As a hybrid solution that combines laser and conventional machining, laser-assisted machining (LAM) can improve the machinability of metal parts by preheating materials using a laser beam as an external heat source to soften the workpiece material [186,187]. The research conducted by Gao, Bermingham, Dargusch, and Matthew et al. [188,189,190,191] provided a better understanding of the influence of cutting parameters on tool wear and surface quality and offered valuable insights into the complexities of machining titanium alloys at elevated temperature. Navas et al. [192] investigated the machinability of Inconel 718 and found that laser heating led to a significant reduction in the work-hardening effect, and the tensile and yield strength of Inconel 718 dropped when the temperatures on the shear zone were around 600–650 °C. Laser heating can result in reductions in cutting force if proper feed rates are applied. Similarly, Dandekar et al. [27] investigated machining characteristics during the hybrid manufacturing of a titanium workpiece with laser-assisted machining and cryogenic cooling of tools. They found that the specific cutting energy was reduced by up to 20% and the surface roughness was reduced by 30% compared to conventional machining. In the hybrid manufacturing process, cryogenic cooling led to lower temperatures in the tool–chip interface to reduce the cutting temperature and tool wear. The surface quality was also improved because of the lower friction between the tool flank face and the workpiece.

In LAM, the softening effect of the machined material by local heating is a critical factor that reduces the cutting force and tool wear. However, during the additive and subtractive hybrid manufacturing (ASHM) process, the laser source leads to a build-up of temperature, and the whole workpiece is heated. It results in a temperature field which is different from local heating, and the machining response (such as machining force, surface integrity, and tool wear) is impacted during successive machining processes [193,194]. Li et al. [180] investigated the influence of temperature build-up on the machinability of DMD-produced Ti-6Al-4V during the ASHM process. The heating device was designed to conduct the milling experiments at a specific temperature. The cutting temperature rose with the rise in the preheating temperature, which reduced the material flow stress. The thermal softening effect was counteracted by the work-hardening effect when the preheating temperature was less than 300 °C. With the further rise in the preheating temperature, the enhanced thermal softening effect led to significantly reduced cutting forces.

In another study, Moritz et al. [194] analysed the influence of cryogenic milling on the machinability of Ti-6Al-4V components fabricated by LMD by adopting carbon dioxide as the coolant to evaluate the surface integrity and tool wear. It was found that cryogenic machining could cause lower surface roughness and smaller tool wear, leading to contamination-free surfaces compared to other machining methods. The result shows that hybrid manufacturing consisting of LMD and cryogenic milling provides significant advantages for the final parts when machining additively manufactured Ti-6Al-4V. Likewise, to further improve the machinability of additively manufactured parts using ASHM, Du et al. [195] presented a creative method that integrated eddy current detection (ECD) into the ASHM process for defect detection/removal of additively manufactured metal parts during the manufacturing process. This method provides the ability for in-process defect detection/removal without the needs for advanced equipment and offers an effective way to further improve the quality of additively manufactured parts of complex shapes.

## 5. Future Development and Conclusions

PBF and DED processes have been progressively used to produce complex alloy parts with near-net shapes. As shown in Table 1, a lot of research has been conducted in the past ten years on powder bed fusion (both SLM and EBM) and directed energy deposition processes. In comparison, there is limited research on wire-based AM processes, such as wire arc additive manufacturing process.

Since some mechanical properties of AM-produced Ti alloys, such as strength and hardness, are significantly larger than those of wrought Ti alloys, the machining of additively manufactured Ti-6Al-4V alloy is a difficult process. The higher cutting force caused by the higher hardness and higher yield strength of additively manufactured parts leads to a higher cutting temperature and severe tool wear, which not only impact the final surface quality of the additively manufactured parts, but also result in premature tool failure. The surface roughness of additively manufactured Ti parts is larger than that of wrought Ti parts. Similar cooling methods that are suitable for machining wrought Ti alloys, such as cryogenic cooling and MQL, are used when machining additively manufactured Ti alloy to reduce the cutting temperature and tool wear. Since the material properties of additively manufactured parts, including microstructural anisotropy and porosity, change with the building orientations, post-processing processes, such as HIP and heat treatment, can be used to alter the microstructural characteristics of additively manufactured components. Microstructural anisotropy of additively manufactured parts results in an unstable manufacturing process because of the fluctuation in cutting force, which further leads to unpredictable tool wear and poor surface integrity. It has been reported that the machining direction has a close relationship with the build-up orientation. The cutting force is affected by different build directions due to the orientation of α-grain boundaries. The best machining performances can be achieved when the machining processes are perpendicular to the build-up direction. However, the relationship between microstructural characteristics and machinability, as well as the fundamental mechanisms, is still not clearly understood. Further exploration of the relationship between machining characteristics and printing strategies is needed.

The additive and subtractive hybrid manufacturing process combines the benefits of both precision subtractive machining and additive manufacturing. It is a promising method with significant potentials for manufacturing additively manufactured Ti parts with complex geometries, which previously have not been possible. In the hybrid machining process, the heat source such as the laser beam, which can be applied to preheat and soften the material, reduces the hardness, yield strength, and tensile strength of the material, leading to an easier machining process and significant enhancement in the machinability of additively manufactured Ti alloy workpieces. In LAM, the softening effect of the machined material by local heating is a critical factor that decreases the cutting force and reduces tool wear. However, during the ASHM process, the whole workpiece is heated, thus inducing a different temperature field in comparison to the local heating method, and may affect the machining characteristics of additively manufactured Ti workpieces.

Additionally, machining strategies for conventional turning and milling processes are used in situ in the hybrid additive and subtractive manufacturing process. Nevertheless, these strategies may not be directly suitable for actual machining applications, for example, additively manufactured Ti-6Al-4V parts with specialised geometry, such as thin walls, and specially shaped overhang features. Hence, more studies are expected in this field. Additionally, conventional cutting tools are used in most machining processes. There is limited research on the design of new cutting tools for the cutting of additively manufactured Ti components. To obtain minimal tool wear and better surface quality, there is a pressing need for more in-depth research on hybrid manufacturing additively manufactured Ti alloys, including machining-induced defects on workpieces and design of new tools that are customised for the in situ machining of additively manufactured parts.

## Figures and Tables

**Table 1:** Recent publications on machining of additively manufactured Ti-6Al-4V alloy.

Reference	AM Process	Machining Process	Investigated Machining Characteristics	Year of Publication
[18]	SLM	Turning	Cutting force, tool wear, and surface roughness	2016
[97]	SLM	Turning	Tool wear and surface integrity	2016
[96]	SLM	Turning	Cutting force and surface integrity	2017
[98]	SLM	Milling	Cutting force and surface integrity	2019
[121]	SLM	Milling	Cutting force and tool wear	2020
[120]	SLM	Milling	Cutting force, tool wear, and surface roughness	2022
[107]	EBM	Turning	Tool wear, surface integrity, and chip morphology	2017
[106]	EBM	Turning	Surface integrity	2019
[35]	EBM	Milling	Surface integrity	2018
[196]	EBM	Milling	Cutting force and surface quality	2020
[112]	DED	Turning	Surface integrity and tool wear	2018
[131]	DED	Turning	Cutting force, surface finish, and tool wear	2018
[197]	DED	Milling	Subsurface deformation	2022
[198]	EBM/DMLS	Turning	Tool wear	2016
[119]	EBM/DMLS	Turning	Tool wear	2017
[110]	DMD	Turning	Surface integrity	2016
[109]	DMLS	Turning	Surface integrity	2016
[129]	EBM	Turning	Tool wear	2015
[137]	SLM	Turning	Cutting force, chip morphology, and microstructural characteristics	2017
[159]	PBF-LB	Milling	Tool wear, surface roughness, and chip morphology	2020
[123]	SLM	Milling	Tool wear, residual stresses, and surface quality	2020
[180]	DMD	Milling	Cutting force and tool wear	2020
[199]	SLM	Milling	Cutting force, tool wear, and chip morphology	2020
[125]	DMLS	Milling	Cutting force, tool wear, chip morphology, and surface integrity	2020
[200]	EBM	Milling	Surface finish, tool wear, and microstructure	2020
[201]	PBF-LB	Milling	Cutting force, surface topography, and chip morphology	2021
[202]	PBF-LB	Turning	Microstructure and surface topography	2021
[203]	PBF-LB	Milling	Surface quality and chip morphology	2021
[204]	SLM	Milling	Surface integrity	2022
[205]	SLM	Turning	Tool wear and surface characteristics	2022
[206]	SLM	Turning	Cutting force, surface integrity, and tool wear	2022
[207]	SLM	Turning	Cutting force, surface quality, and tool wear	2022
[208]	SLM	Milling	Cutting force and surface roughness	2022
[209]	DMLS	Turning	Tool wear, surface roughness, and chip morphology	2022
[210]	DMLS	Milling	Milling force, residual stresses, and subsurface plastic deformation	2022
[211]	EBM	Turning	Cutting force and surface roughness	2022

## Data Availability

Not applicable.
